# Needle bearing endurance test data for early fault detection during oscillating movements

**DOI:** 10.1016/j.dib.2026.113268

**Published:** 2026-09-16

**Authors:** Timur Seferli, Jens Windelberg, Andreas Bierig

**Affiliations:** German Aerospace Center (DLR) – Institute of Flight System, Lilienthalplatz 7, 38108, Braunschweig, Germany

**Keywords:** Needle bearings, Oscillation, Condition monitoring, Test rig

## Abstract

In aviation, there is a transition to more electric aircraft (MEA) for commercial aircraft, as electric powered components promise an increase in operational efficiency. These components are being integrated into the primary and secondary flight control system. The adaptation of electromechanical actuators (EMAs), for instance, can enhance maintainability, reliability and reduce the overall aircraft weight. However, the service life of such an actuator depends on the failure mechanisms of its components. During operation, bearings are critical components because they experience short-stroke movements that may lead to early failure due to fretting corrosion. Under these conditions, the amplitude of the oscillation, the frequency and the applied load vary. To ensure the safe and reliable operation of EMAs, it is necessary to implement an early fault detection mechanism and to improve oscillating bearing lifetime calculation. For accurate prediction of the remaining useful life (RUL), high-fidelity and reliable data are essential. Vibration measurements or analyses of torque signals are frequently used for this purpose. Thus, this data article presents a dataset of eight independent run-to-failure experimental data. The performance of needle bearings was tested using different lubricants. In these tests, the bearings were radial preloaded with 9 to 19.4 kN, the oscillation angle varied between 10° and 20°, and frequencies of 5 Hz and 10 Hz were applied. This article provides measurements of acceleration, temperature, positional displacement, torque fluctuation, and radial movement dynamics obtained from oscillating needle bearings. This dataset serves as a valuable resource to improve the understanding of signal characteristics associated with bearing degradation and fault progression. The results from different tested initial parameters can be leveraged to develop and validate RUL predictive models in various industrial and aerospace engineering applications.

Specifications Table

**Table:** 

Subject	Engineering & Materials science	
Specific subject area	Experimental data of oscillating needle bearings with typical movements in the aviation or wind industry. The data are raw data for the realisation of condition monitoring models.	
Type of data	Raw Data, Datasets in “.mat” format, Figures (.jpg)	
Data collection	A test rig was specially designed for lifetime tests for bearings. Two needle bearings were tested simultaneously so that two sets of measurements could be analysed, with each bearing equipped with its own sensors. This approach ensures identical environmental conditions. The bearings were rotated by controlling the inner ring angle commanding a sinusoidal profile with a given amplitude and frequency. The data acquisition system consists of two accelerometers (acceleration limit ±50 g), two radial load mechanisms (up to 50 kN), two distance sensors (0 to 3 mm), three temperature sensors (one for each needle bearing, one for the ambient temperature) and one encoder. The needle bearing under test was fitted in a bearing shell where the sensors are mounted. A load is applied radially to the needle bearing. Under the preload, the bearings are tested with different starting parameters (lubrication, frequency or angle). The applied oscillating movements simulate typical bearing movements in the aviation or wind industry. This paper includes 8 independent datasets of oscillating needle bearings. Both bearings are mounted on a shaft. The oscillation angle is specified and controlled by a single encoder. Consequently, both bearings follow the same oscillation. However, each bearing is equipped with its own force, acceleration, distance, and temperature sensor, which operate independently of one another. Different conditions such as lubrication, amplitude of the oscillation, frequency and a radial applied load were tested. The key measurement criterion is to collect a broad spectrum of measurement signals in order to recognize degradations at an early stage. Acceleration, distance, force, temperature, torsion angle and torque measurements were therefore recorded with the following sample rates:	
		Sample rate
	Acceleration	10,000 Hz
	Distance	10,000 Hz
	Force	100 Hz
	Temperature	10 Hz
	Oscillation angle	1000 Hz
	Torque	10,000 Hz
Data source location	DLR (German Aerospace Center)	
	Institute of Flight Systems	
	Condition Monitoring Research Group	
	Braunschweig, Germany	
	Latitude: 52.3151 | Longitude: 10.5640	
Data accessibility	Repository name: zenodo	
	Data identification number: 10.5281/zenodo.20742912	
	Direct URL to data: https://doi.org/10.5281/zenodo.20742913	
Related research article	Seferli T., Dreyer N., Kowalski R., Bierig A. (2025). Experimental investigation of oscillating bearings in EMA by using accelerated lifetime tests. In J.-C. Mare (Ed.), Proceedings of the tenth International Conference on Recent Advances in Aerospace Actuation Systems and Components (pp. 93–99). INSA de Toulouse.	

## 1 Value of the Data

• This dataset fills the gap in measurements of oscillating bearings, as continuous rotation is the normal type of movement in many applications. In combination with studies such as [1,2], service-life calculation models can be extended.
• This dataset consists of run-to-failure measurements for oscillating high-performance needle bearings. Various operating conditions such as variations in lubrication, preload or frequency were tested.
• The dataset can be used to conduct studies on the health monitoring of oscillating needle bearings, as motor torque, acceleration signals, torsion angles, radial movements, preload forces and temperature changes were recorded over the service life. Multiple studies [[3], [4], [5]] demonstrate that this sensor data are suitable for health monitoring.
• The dataset captures both damage-free (beginning of tests) and degraded (failure-state) conditions, offering valuable data for fault detection and prognosis models in the aviation or wind industry.

## 2 Objective

This data article is intended to support research to determine the failure mode of bearings in an oscillating motion. In reality, many components perform oscillating movements, such as in the aviation industry (e.g. bearings in flight control actuators) or in the wind industry (e.g. bearings for individual pitch control [6]).

During a continuous rotational movement, surface fatigue is the predominant wear mechanism [1,7]. However, during an oscillating movement other failure mechanism such as fretting and false brinelling occur and significantly reduce service life [1,7]. In particular, DIN ISO 3408–5 is used to calculate the service life of bearings, but it is applicable only under certain conditions, such as continuous rotation and ideal lubrication [1,8]. Therefore, component tests are essential for validating the service life (Fig. 1).

**Fig. 1:**
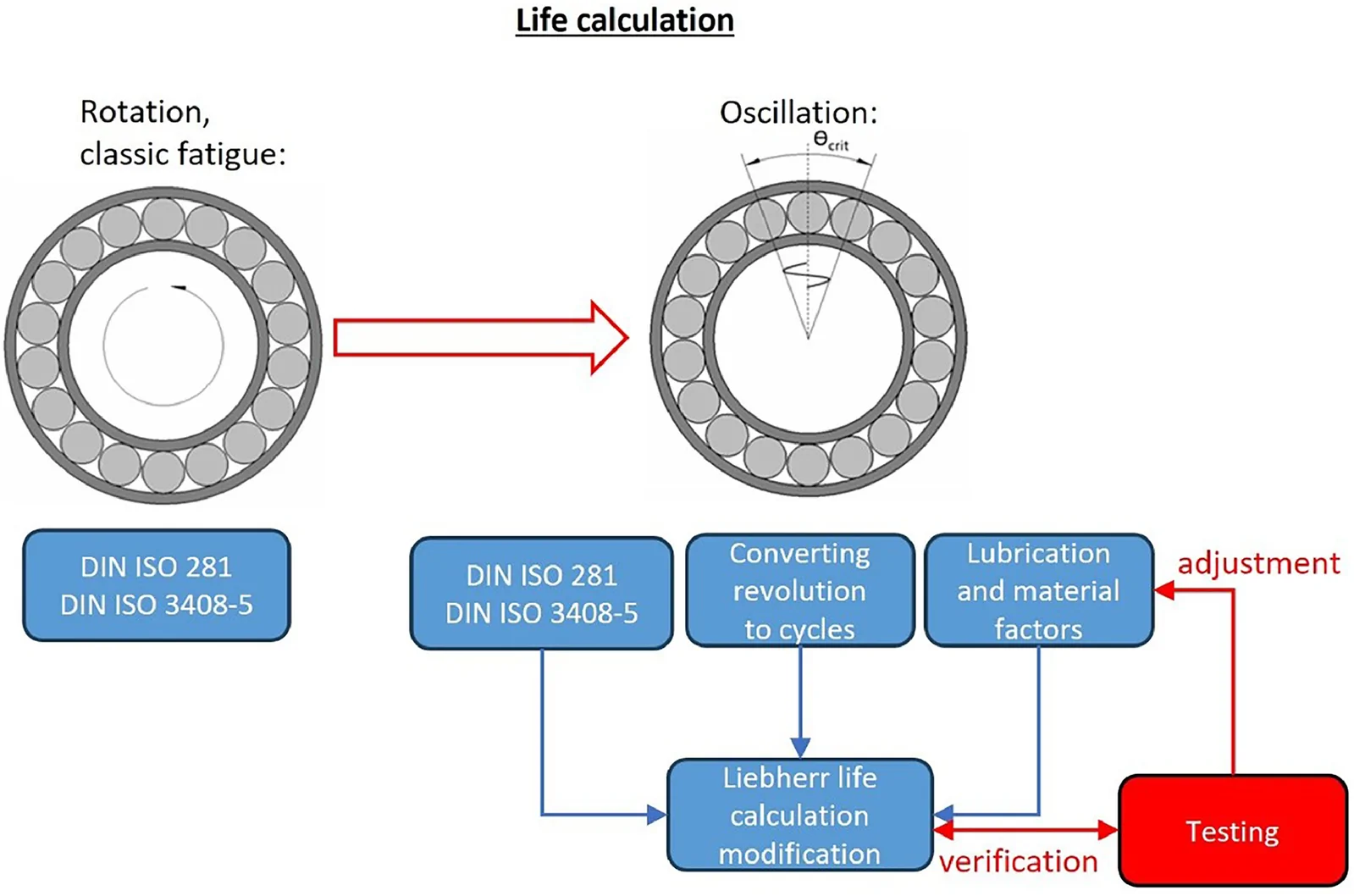
Modification of life calculation for oscillating bearings [1].

This article presents test data from an oscillating high-performance needle bearing. Various studies [[3], [4], [5]] were reviewed to identify the required sensor data for service-life prognosis. Consequently, measurements of acceleration, distance, force, temperature, torsion angle, and torque were recorded. The knowledge gained can be used in the development of service-life predictions or service-life models. No service-life forecasting is performed in this study.

In this study, eight independent oscillatory tests were performed under different staring conditions. In each test, two bearings were examined to ensure that the environmental conditions were identical. The objective was to determine the influence of the test conditions on service life.

This paper is structured as follows: Section 2.1 illustrates the needle bearing and specifies its geometric dimensions. Section 3 defines the measurement parameters and presents the measurement data schematically. Section 4 shows the test rig used and illustrates the setup and positioning of the sensors.

### 2.1 Characteristics of the needle bearing

A highly loaded needle bearing from an actuator was tested [9]. During the test, the bearing has two contact lines. The inner contact line is between a shaft and the needle. The outer contact line is between the needle and an outer shell. The outer shell has been specially designed to enable the attachment of sensors (Fig. 2).

**Fig. 2:**
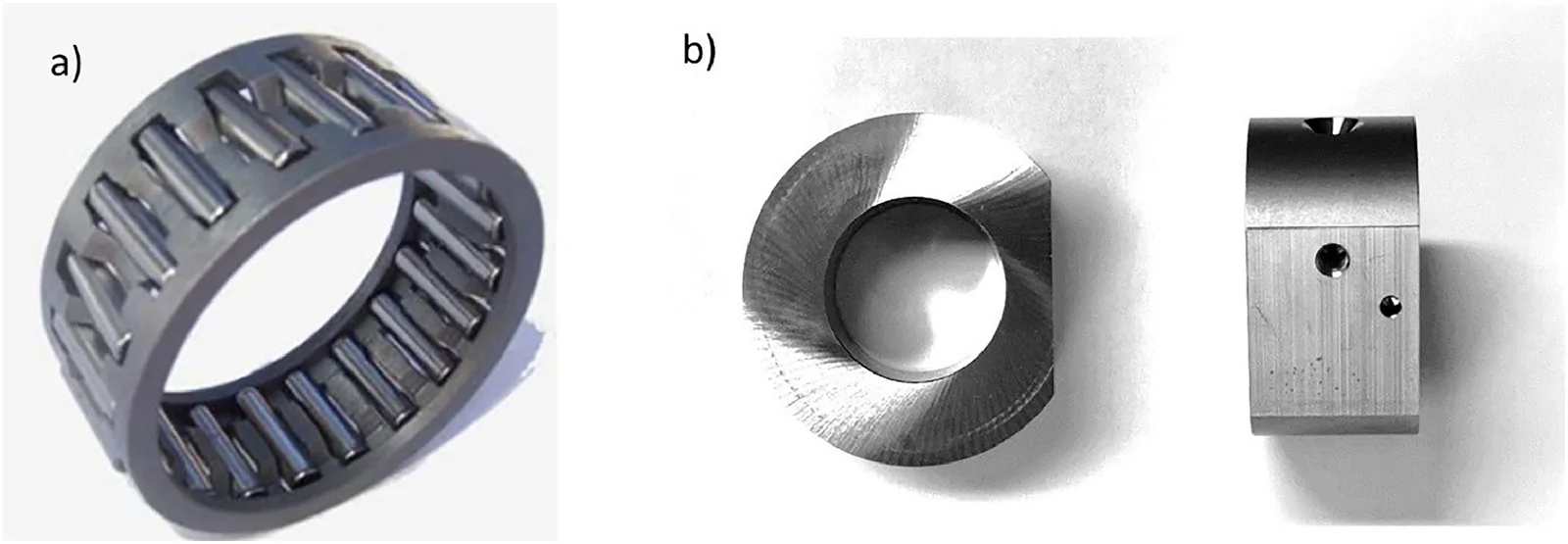
a) Needle bearing ring and b) bearing shell.

Two needle bearings are tested per test. The sensors connected to needle bearing 1 have the suffix _1 and the sensors connected to needle bearing 2 have the suffix _2. All relevant geometric dimensions and physical properties of the bearing are documented in Table 1.

**Table 1:** Characteristics of the tested needle bearing [10].

Parameter	Value
Number of needles	12
Needle diameter	3.00 mm
Needle pitch diameter	18.00 mm
Component width	21.00 mm
Basic dynamic load rating, radial	19,400 N
Basic static load rating, radial	24,300 N

## 3 Data Description

The dataset contains eight independent measurements of two needle bearings each. Two needle bearings are always mounted on a single shaft so that they are subjected to identical external environmental conditions. The oscillation angle is controlled by a single encoder. In addition, each bearing is equipped with dedicated acceleration, distance, and temperature sensors, a load cell, and an oil reservoir. Consequently, the needle bearings exhibit the same oscillation but have independent sensors. A run-to-failure test is conducted until the needle bearings fail. Because the test cannot be interrupted to remove and inspect the needle bearings, it is terminated if damage is detected. The needle bearings are then disassembled, and their running surfaces are examined for damage (Fig. 4).

All recordings end when the bearing has failed. The failure criterion is based on fulfilment of its (bearing) intended function and is dependent on the final application. Here it was decided that an increase in friction torque (>50Nm), induced accelerations above 50 g, exceedance of temperature above 50 °C would violate typical functional or performance requirements. All limits are intentionally set to high levels (probably too high for real world applications) in order to capture the whole degradation for real world applications. The data should thus be truncated by the user when their specific failure criterion is met. Since these values were set at very high levels, all tests were terminated manually because the oscillation angle fell substantially below the initial value specified in Table 2.

**Table 2:** Test parameters.

Test number	Kinematic viscosity at 40 °C	Sine wave amplitude	Radial preload	Frequency
1	6.75 mm²/s	10°	9 kN	10 Hz
2	17.4 mm²/s	10°	9 kN	10 Hz
3	13 mm²/s	10°	9 kN	10 Hz
4	no lubrication	20°	9 kN	10 Hz
5	no lubrication	10°	9 kN	10 Hz
6	no lubrication	10°	18.7 kN	5 Hz
7	no lubrication	10°	19.4 kN	5 Hz
8	no lubrication	10°	19.4 kN	10 Hz

During the test, motor torque (in Nm), acceleration values (in g), oscillation angle (in °), thermal measurements (in °C) at the outer contact point and a radial displacement (in mm) of the bearings were recorded (Fig. 5). All test conditions and configurated parameter can be found in Table 2.

A total of three lubricants were tested to determine the effect of the lubricants on the needle bearings, allowing for a direct comparison with baseline measurements taken from tests conducted without any lubrication.

The radial preload was set once at room temperature before the test begins. The amplitude of the oscillation was checked before the start of the test and under preload. During the test the changes in radial force was recorded.

For data management, the same approach as in [11,12] is adopted. Recordings are initiated at regular intervals rather than continuously. In bearing run-to-failure tests, rapid failure is not expected; therefore, other sources such as [12], record only one second every ten minutes. Preliminary testing showed that the tests lasted several million cycles and that no significant change in the degradation occurred within 15 min. At a test frequency of 10 Hz, 100 cycles correspond to a 10 s interval. Given that the expected number of cycles is in the millions, four measurements (400 cycles) can therefore be performed in one hour. Due to the large number of measured data (11 sensors with a sampling rate of up to 10 kHz), it was chosen to record in a time interval of ten seconds every 15 min (Fig. 3). The datasets were stored in the standard MATLAB format, ".mat".

**Fig. 3:**
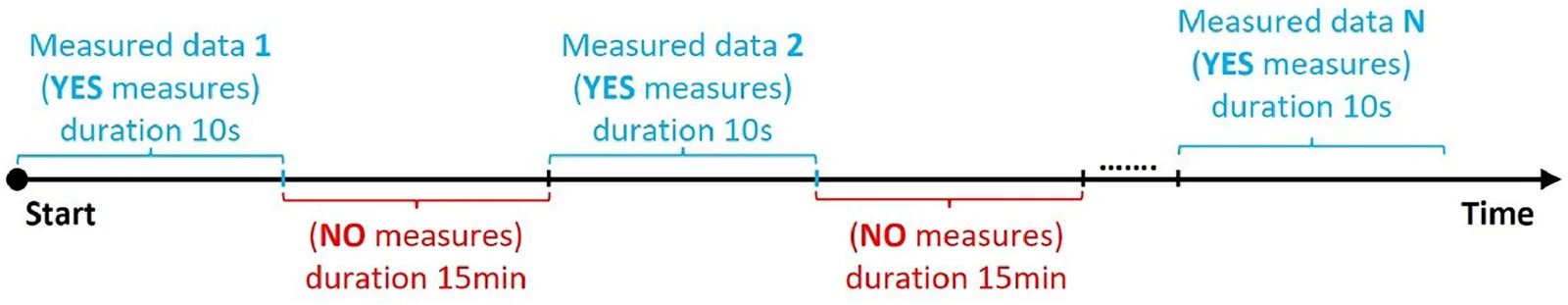
Test strategy. The test starts with a recording of 10 s. After 15 min the next recording will be initiated. The experiment ends with N measured data after the experiment is stopped.

Each measured data was summarised in a single column. The individual sensor readings are provided as a matrix, where the first column represents the time vector (in seconds) and the second column contains the corresponding sensor measurements (Table 3). To minimise the data volume in the matrix, the dataset tests 1,3 and 5 were split.

**Table 3:** Needle bearing dataset.

Test number	File name (.mat)	Remark
1	Test1_Oil3_10-9-10_Data_1_to_285_Position_deg	During measurement data **286–-290** and **893–-939** a screw of the encoder came loose, resulting in corrupted data. The recordings were therefore removed.
	Test1_Oil3_10-9-10_Data_291_to_892_Position_deg	In addition, the distance sensors experienced a fault in the timespan from of measurement data **893** to **939**. Therefore, no data was recorded.
	Test1_Oil3_10-9-10_Data_940_to_1326_Position_deg	
	Test1_Oil3_10-9-10_Data_1_to_285_Torque	
	Test1_Oil3_10-9-10_Data_291_to_892_Torque	
	Test1_Oil3_10-9-10_Data_940_to_1326_Torque	
	Test1_Oil3_10-9-10_Data_1_to_285_Ambient_Temp	
	Test1_Oil3_10-9-10_Data_291_to_892_Ambient_Temp	
	Test1_Oil3_10-9-10_Data_940_to_1326_Ambient_Temp	
	Needle_1:	
	Test1_Oil3_10-9-10_Data_1_to_285_Acc_Sensor_1	
	Test1_Oil3_10-9-10_Data_291_to_892_Acc_Sensor_1	
	Test1_Oil3_10-9-10_Data_940_to_1326_Acc_Sensor_1	
	Test1_Oil3_10-9-10_Data_1_to_285_Distance_Sensor_1	
	Test1_Oil3_10-9-10_Data_291_to_892_Distance_Sensor_1	
	Test1_Oil3_10-9-10_Data_1_to_285_Force_Sensor_1	
	Test1_Oil3_10-9-10_Data_291_to_892_Force_Sensor_1	
	Test1_Oil3_10-9-10_Data_940_to_1326_Force_Sensor_1	
	Test1_Oil3_10-9-10_Data_1_to_285_Temp_Sensor_1	
	Test1_Oil3_10-9-10_Data_291_to_892_Temp_Sensor_1	
	Test1_Oil3_10-9-10_Data_940_to_1326_Temp_Sensor_1	
	Needle_2:	
	Test1_Oil3_10-9-10_Data_1_to_285_Acc_Sensor_2	
	Test1_Oil3_10-9-10_Data_291_to_892_Acc_Sensor_2	
	Test1_Oil3_10-9-10_Data_940_to_1326_Acc_Sensor_2	
	Test1_Oil3_10-9-10_Data_1_to_285_Distance_Sensor_2	
	Test1_Oil3_10-9-10_Data_291_to_892_Distance_Sensor_2	
	Test1_Oil3_10-9-10_Data_1_to_285_Force_Sensor_2	
	Test1_Oil3_10-9-10_Data_291_to_892_Force_Sensor_2	
	Test1_Oil3_10-9-10_Data_940_to_1326_Force_Sensor_2	
	Test1_Oil3_10-9-10_Data_1_to_285_Temp_Sensor_2	
	Test1_Oil3_10-9-10_Data_291_to_892_Temp_Sensor_2	
	Test1_Oil3_10-9-10_Data_940_to_1326_Temp_Sensor_2	
2	Test2_Oil2_10-9-10_Data_1_to_1032_Position_deg	
	Test2_Oil2_10-9-10_Data_1_to_1032_Torque	
	Test2_Oil2_10-9-10_Data_1_to_1032_Ambient_Temp	
	Needle_1:	
	Test2_Oil2_10-9-10_Data_1_to_1032_Acc_Sensor_1	
	Test2_Oil2_10-9-10_Data_1_to_1032_Distance_Sensor_1	
	Test2_Oil2_10-9-10_Data_1_to_1032_Force_Sensor_1	
	Test2_Oil2_10-9-10_Data_1_to_1032_Temp_Sensor_1	
	Needle_2:	
	Test2_Oil2_10-9-10_Data_1_to_1032_Acc_Sensor_2	
	Test2_Oil2_10-9-10_Data_1_to_1032_Distance_Sensor_2	
	Test2_Oil2_10-9-10_Data_1_to_1032_Force_Sensor_2	
	Test2_Oil2_10-9-10_Data_1_to_1032_Temp_Sensor_2	
3	Test3_Oil1_10-9-10_Data_1_to_1000_Position_deg	The test was stopped after recording data **765** (at second 695,250) to be able to refill the oil dripper.
	Test3_Oil1_10-9-10_Data_1001_to_2000_Position_deg	The test was briefly paused after the **2552**nd measurement (at second 2,321,420) to check the sensors.
	Test3_Oil1_10-9-10_Data_2001_to_3000_Position_deg	
	Test3_Oil1_10-9-10_Data_3001_to_4000_Position_deg	
	Test3_Oil1_10-9-10_Data_4001_to_4951_Position_deg	
	Test3_Oil1_10-9-10_Data_1_to_1000_Torque	
	Test3_Oil1_10-9-10_Data_1001_to_2000_Torque	
	Test3_Oil1_10-9-10_Data_2001_to_3000_Torque	
	Test3_Oil1_10-9-10_Data_3001_to_4000_Torque	
	Test3_Oil1_10-9-10_Data_4001_to_4951_Torque	
	Test3_Oil1_10-9-10_Data_1_to_1000_Ambient_Temp	
	Test3_Oil1_10-9-10_Data_1001_to_2000_Ambient_Temp	
	Test3_Oil1_10-9-10_Data_2001_to_3000_Ambient_Temp	
	Test3_Oil1_10-9-10_Data_3001_to_4000_Ambient_Temp	
	Test3_Oil1_10-9-10_Data_4001_to_4951_Ambient_Temp	
	Needle_1:	
	Test3_Oil1_10-9-10_Data_1_to_1000_Acc_Sensor_1	
	Test3_Oil1_10-9-10_Data_1001_to_2000_Acc_Sensor_1	
	Test3_Oil1_10-9-10_Data_2001_to_3000_Acc_Sensor_1	
	Test3_Oil1_10-9-10_Data_3001_to_4000_Acc_Sensor_1	
	Test3_Oil1_10-9-10_Data_4001_to_4951_Acc_Sensor_1	
	Test3_Oil1_10-9-10_Data_1_to_1000_Distance_Sensor_1	
	Test3_Oil1_10-9-10_Data_1001_to_2000_Distance_Sensor_1	
	Test3_Oil1_10-9-10_Data_2001_to_3000_Distance_Sensor_1	
	Test3_Oil1_10-9-10_Data_3001_to_4000_Distance_Sensor_1	
	Test3_Oil1_10-9-10_Data_4001_to_4951_Distance_Sensor_1	
	Test3_Oil1_10-9-10_Data_1_to_1000_Force_Sensor_1	
	Test3_Oil1_10-9-10_Data_1001_to_2000_Force_Sensor_1	
	Test3_Oil1_10-9-10_Data_2001_to_3000_Force_Sensor_1	
	Test3_Oil1_10-9-10_Data_3001_to_4000_Force_Sensor_1	
	Test3_Oil1_10-9-10_Data_4001_to_4951_Force_Sensor_1	
	Test3_Oil1_10-9-10_Data_1_to_1000_Temp_Sensor_1	
	Test3_Oil1_10-9-10_Data_1001_to_2000_Temp_Sensor_1	
	Test3_Oil1_10-9-10_Data_2001_to_3000_Temp_Sensor_1	
	Test3_Oil1_10-9-10_Data_3001_to_4000_Temp_Sensor_1	
	Test3_Oil1_10-9-10_Data_4001_to_4951_Temp_Sensor_1	
	Needle_2:	
	Test3_Oil1_10-9-10_Data_1_to_1000_Acc_Sensor_2	
	Test3_Oil1_10-9-10_Data_1001_to_2000_Acc_Sensor_2	
	Test3_Oil1_10-9-10_Data_2001_to_3000_Acc_Sensor_2	
	Test3_Oil1_10-9-10_Data_3001_to_4000_Acc_Sensor_2	
	Test3_Oil1_10-9-10_Data_4001_to_4951_Acc_Sensor_2	
	Test3_Oil1_10-9-10_Data_1_to_1000_Distance_Sensor_2	
	Test3_Oil1_10-9-10_Data_1001_to_2000_Distance_Sensor_2	
	Test3_Oil1_10-9-10_Data_2001_to_3000_Distance_Sensor_2	
	Test3_Oil1_10-9-10_Data_3001_to_4000_Distance_Sensor_2	
	Test3_Oil1_10-9-10_Data_4001_to_4951_Distance_Sensor_2	
	Test3_Oil1_10-9-10_Data_1_to_1000_Force_Sensor_2	
	Test3_Oil1_10-9-10_Data_1001_to_2000_Force_Sensor_2	
	Test3_Oil1_10-9-10_Data_2001_to_3000_Force_Sensor_2	
	Test3_Oil1_10-9-10_Data_3001_to_4000_Force_Sensor_2	
	Test3_Oil1_10-9-10_Data_4001_to_4951_Force_Sensor_2	
	Test3_Oil1_10-9-10_Data_1_to_1000_Temp_Sensor_2	
	Test3_Oil1_10-9-10_Data_1001_to_2000_Temp_Sensor_2	
	Test3_Oil1_10-9-10_Data_2001_to_3000_Temp_Sensor_2	
	Test3_Oil1_10-9-10_Data_3001_to_4000_Temp_Sensor_2	
	Test3_Oil1_10-9-10_Data_4001_to_4951_Temp_Sensor_2	
4	Test4_NoOil_20-9-10_Data_1_to_172_Position_deg	
	Test4_NoOil_20-9-10_Data_1_to_172_Torque	
	Test4_NoOil_20-9-10_Data_1_to_172_Ambient_Temp	
	Needle_1:	
	Test4_NoOil_20-9-10_Data_1_to_172_Acc_Sensor_1	
	Test4_NoOil_20-9-10_Data_1_to_172_Distance_Sensor_1	
	Test4_NoOil_20-9-10_Data_1_to_172_Force_Sensor_1	
	Test4_NoOil_20-9-10_Data_1_to_172_Temp_Sensor_1	
	Needle_2:	
	Test4_NoOil_20-9-10_Data_1_to_172_Acc_Sensor_2	
	Test4_NoOil_20-9-10_Data_1_to_172_Distance_Sensor_2	
	Test4_NoOil_20-9-10_Data_1_to_172_Force_Sensor_2	
	Test4_NoOil_20-9-10_Data_1_to_172_Temp_Sensor_2	
5	Test5_NoOil_10-9-10_Data_1_to_1000_Position_deg	The test was briefly paused after the **669**th measurement (at second 607,890) to check the sensors.
	Test5_NoOil_10-9-10_Data_1001_to_1843_Position_deg	
	Test5_NoOil_10-9-10_Data_1_to_1000_Torque	
	Test5_NoOil_10-9-10_Data_1001_to_1843_Torque	
	Test5_NoOil_10-9-10_Data_1_to_1000_Ambient_Temp	
	Test5_NoOil_10-9-10_Data_1001_to_1843_Ambient_Temp	
	Needle_1:	
	Test5_NoOil_10-9-10_Data_1_to_1000_Acc_Sensor_1	
	Test5_NoOil_10-9-10_Data_1001_to_1843_Acc_Sensor_1	
	Test5_NoOil_10-9-10_Data_1_to_1000_Distance_Sensor_1	
	Test5_NoOil_10-9-10_Data_1001_to_1843_Distance_Sensor_1	
	Test5_NoOil_10-9-10_Data_1_to_1000_Force_Sensor_1	
	Test5_NoOil_10-9-10_Data_1001_to_1843_Force_Sensor_1	
	Test5_NoOil_10-9-10_Data_1_to_1000_Temp_Sensor_1	
	Test5_NoOil_10-9-10_Data_1001_to_1843_Temp_Sensor_1	
	Needle_2:	
	Test5_NoOil_10-9-10_Data_1_to_1000_Acc_Sensor_2	
	Test5_NoOil_10-9-10_Data_1001_to_1843_Acc_Sensor_2	
	Test5_NoOil_10-9-10_Data_1_to_1000_Distance_Sensor_2	
	Test5_NoOil_10-9-10_Data_1001_to_1843_Distance_Sensor_2	
	Test5_NoOil_10-9-10_Data_1_to_1000_Force_Sensor_2	
	Test5_NoOil_10-9-10_Data_1001_to_1843_Force_Sensor_2	
	Test5_NoOil_10-9-10_Data_1_to_1000_Temp_Sensor_2	
	Test5_NoOil_10-9-10_Data_1001_to_1843_Temp_Sensor_2	
6	Test6_NoOil_10-18,4-5_Data_1_to_94_Position_deg	The measurement data 1 was started with a slight delay: Recording was started and
	Test6_NoOil_10-18,4-5_Data_1_to_94_Torque	immediately afterwards the drive unit was started.
	Test6_NoOil_10-18,4-5_Data_1_to_94_Ambient_Temp	
	Needle_1:	
	Test6_NoOil_10-18,4-5_Data_1_to_94_Acc_Sensor_1	
	Test6_NoOil_10-18,4-5_Data_1_to_94_Distance_Sensor_1	
	Test6_NoOil_10-18,4-5_Data_1_to_94_Force_Sensor_1	
	Test6_NoOil_10-18,4-5_Data_1_to_94_Temp_Sensor_1	
	Needle_2:	
	Test6_NoOil_10-18,4-5_Data_1_to_94_Acc_Sensor_2	
	Test6_NoOil_10-18,4-5_Data_1_to_94_Distance_Sensor_2	
	Test6_NoOil_10-18,4-5_Data_1_to_94_Force_Sensor_2	
	Test6_NoOil_10-18,4-5_Data_1_to_94_Temp_Sensor_2	
	
7	Test7_NoOil_10-19,4-5_Data_1_to_19_Position_deg	Due to the high temperatures, the test was stopped twice to check the sensors.
	Test7_NoOil_10-19,4-5_Data_1_to_19_Torque	The first sensor check was carried out after the **5**th measurement
	Test7_NoOil_10-19,4-5_Data_1_to_19_Ambient_Temp	(at second 3,650).
	Needle_1:	The second sensor check was carried out after the **13**th measurement (at second 10,930).
	Test7_NoOil_10-19,4-5_Data_1_to_19_Acc_Sensor_1	
	Test7_NoOil_10-19,4-5_Data_1_to_19_Distance_Sensor_1	
	Test7_NoOil_10-19,4-5_Data_1_to_19_Force_Sensor_1	
	Test7_NoOil_10-19,4-5_Data_1_to_19_Temp_Sensor_1	
	Needle_2:	
	Test7_NoOil_10-19,4-5_Data_1_to_19_Acc_Sensor_2	
	Test7_NoOil_10-19,4-5_Data_1_to_19_Distance_Sensor_2	
	Test7_NoOil_10-19,4-5_Data_1_to_19_Force_Sensor_2	
	Test7_NoOil_10-19,4-5_Data_1_to_19_Temp_Sensor_2	
8	Test8_NoOil_10-19,4-10_Data_1_to_205_Position_deg	
	Test8_NoOil_10-19,4-10_Data_1_to_205 _Torque	
	Test8_NoOil_10-19,4-10_Data_1_to_205_Ambient_Temp	
	Needle_1:	
	Test8_NoOil_10-19,4-10_Data_1_to_205_Acc_Sensor_1	
	Test8_NoOil_10-19,4-10_Data_1_to_205 _Distance_Sensor_1	
	Test8_NoOil_10-19,4-10_Data_1_to_205 _Force_Sensor_1	
	Test8_NoOil_10-19,4-10_Data_1_to_205 _Temp_Sensor_1	
	Needle_2:	
	Test8_NoOil_10-19,4-10_Data_1_to_205_Acc_Sensor_2	
	Test8_NoOil_10-19,4-10_Data_1_to_205 _Distance_Sensor_2	
	Test8_NoOil_10-19,4-10_Data_1_to_205 _Force_Sensor_2	
	Test8_NoOil_10-19,4-10_Data_1_to_205 _Temp_Sensor_2	

Eight independent needle bearing tests were conducted. Tests 2, 4 and 8 were run without interruption. Tests 3, 5 and 7 were stopped during the experiment to inspect the sensors. In Test 1, a screw on the encoder became loose: Consequently, the test rig was not operational during measurement files 286–290 and 893–939, and the corresponding recordings were deleted. In addition, a distance-sensor failure occurred during measurement files 893–939, and no recorded data are available for this period.

After the service life test was completed, the bearings were disassembled and inspected for visible damage. Fig. 4 shows the damage on the bearing shell and shaft of needle bearing 2 in Test 7. All eight tests were stopped manually because the oscillation angle had decreased significantly. Table 4 presents a summary of the bearings after testing.

**Fig. 4:**
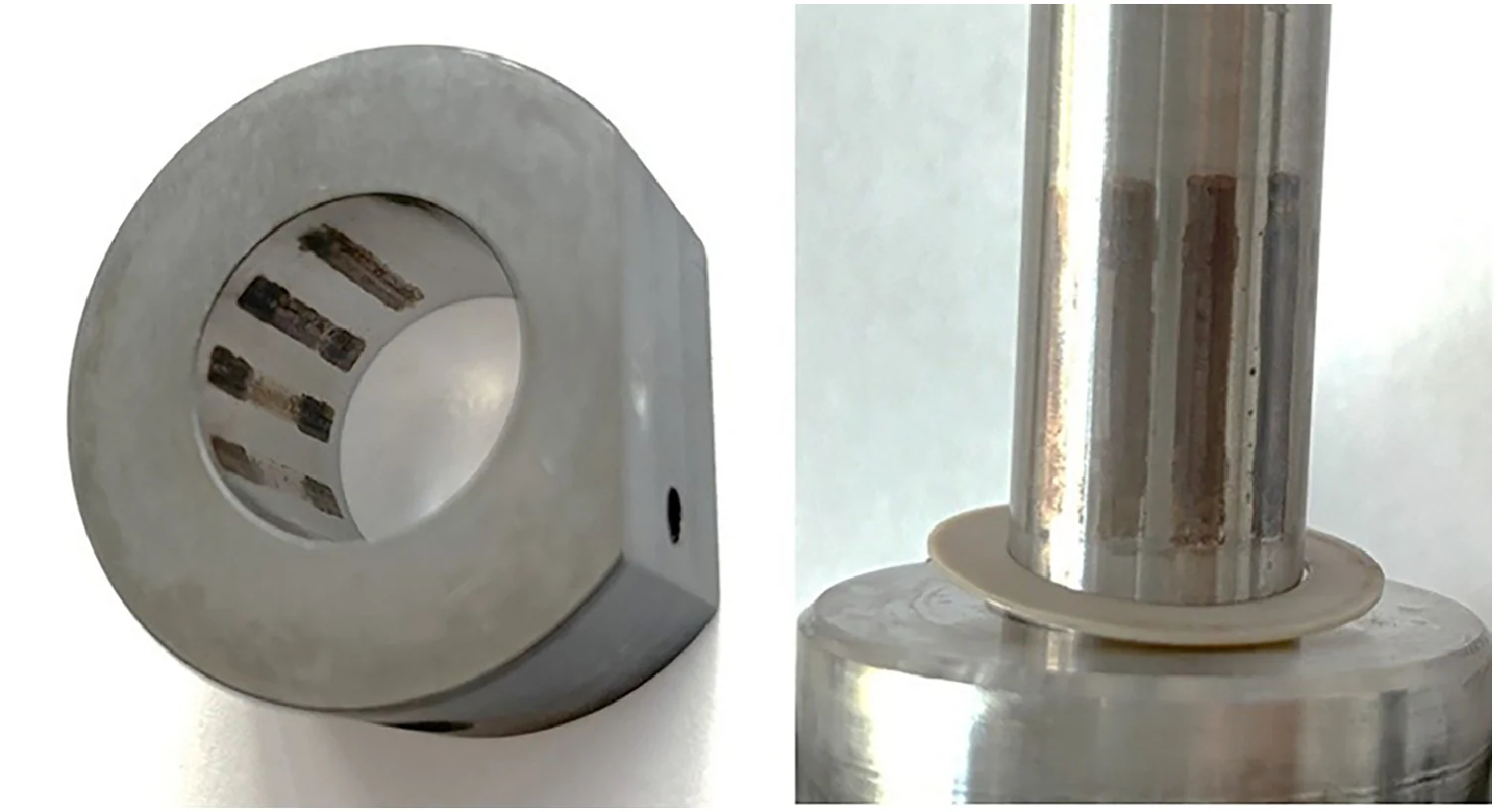
Damage of bearing shell (left) und shaft (right) of needle 7-2.

**Table 4:** Summary of the needle bearing test.

Test number	Is the bearing damaged?		Data interruptions and sensor faults	Recorded cycles
	Needle 1	Needle 2		
1	Yes	Yes	Sensor faults: • Encoder fault (during measurement data 286–290 and 893–939) • Distance sensor fault (during measurement data 893 to 939)	11,575,200
2	Yes	Yes	-	9391,200
3	Yes	Yes	Test was paused: • to refill oil • to check the sensors (After the 765th and 2552nd measurement)	45,054,100
4	Yes	Yes	-	1565,200
5	Yes	Yes	Test was paused: • to check the sensors (After the 669th measurement)	16,771,300
6	Yes	Yes	Measurement data 1 was recorded with a slight delay.	427,700
7	Yes	Yes	Test was paused due to high temperatures: • to check the sensors (after the 5th and 13th measurement)	86,450
8	Yes	Yes	**-**	1865,500

## 4 Experimental Design

A test rig was developed to generate service life test data, which is described in more detail in [13,8]. This test rig was constructed and assembled in the DLR laboratory (Fig. 5). For each experiment, two needle bearings were tested simultaneously. The test rig is powered by an MCS synchronous servo motor from Lenze (model: MCS19J29-RS0B0-B28N-ST5F10N-R0SU [14]), and the torque is transmitted to the shaft through a coupling.

**Fig. 5:**
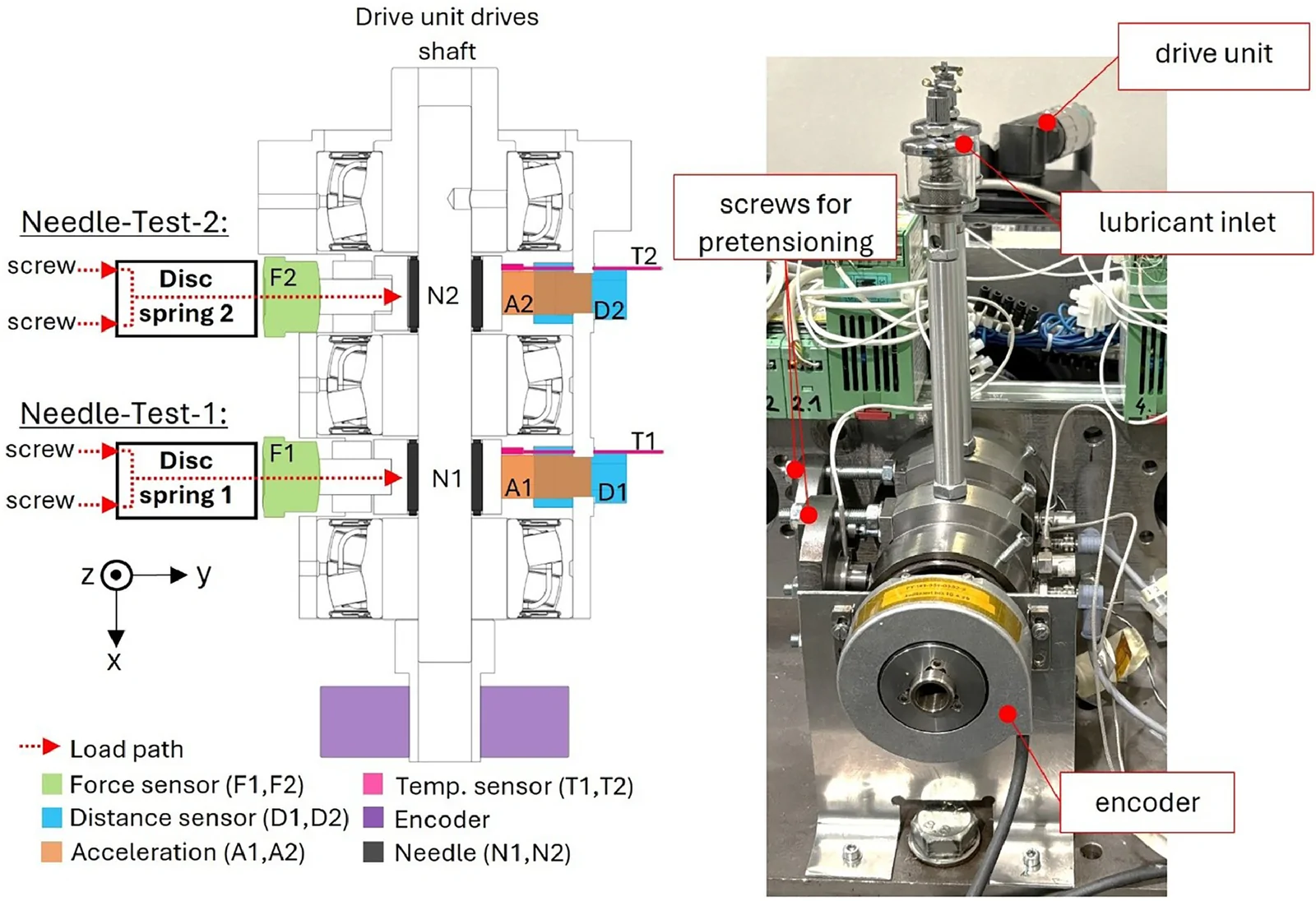
Test rig and sensor placement [8].

A total of two load cells from Burster Präzisionsmesstechnik (model: 8402–6050-N000S000 [15]) are used to measure the applied force in the Y-direction. The change in force is measured at each needle bearing. The force is applied through disc springs and adjusted by screws. It is transmitted directly to the bearing via a pin located between the load cell and the bearing shell.

On the opposite side, an acceleration sensor (Kistler K-Shear: 8702B25M1T05 [16]), an inductive distance sensor (Baumer: IR08.D03L-Q46.UA1T.7SL [17]) and a temperature sensor (Honeywell: HEL-705-U-1–12–00 [18]) are installed for each needle bearing. With the distance sensor, it is possible to measure a movement in the Y-direction of up to 3 mm [17]. In addition to the two temperature sensors on the needle bearings, an extra temperature sensor is used to record the ambient temperature during the experiment. A drip oiler is attached to each needle bearing so that the influence of different lubricants can be analysed.

An encoder (Heidenhain: ERN 180 5000 [19]) is used both to measure the shaft’s angle of rotation and to control it during operation.

Fretting corrosion triggered by lubricant starvation [20] and oscillation under load [21] is assumed the dominant failure mode of oscillating bearings. In order to capture the influence of the parameters individually, only variation of one variable according to Table 2 is carried out per test run. The amplitudes of the oscillation Θ=10° and Θ=20° were chosen because the movement will remain in the range of (Table 5) [22]:

(1) $$\Theta_{dith}<\Theta <\Theta_{crit}$$

**Table 5:** Calculated dithering angle and critical angle for the bearing.

Angle (Θ)	Inner contact line (shaft-needle)	Outer contact line (needle-shell)
Critical angle (*Θ_crit_*)	51.43 °	72°
Dithering angle (*Θ_dith_*)	0.70 °	0.61°

Below the dithering angle false brinelling is expected, while above the critical angle conventional fatigue degradation should predominate. In the range between the dithering angle and the critical angle, fretting corrosion is probably the predominant failure mode [22]. The maximum value for the basic dynamic load rating C*_r_* =19.400 N was not exceeded for the applied force [10].

## Limitations

Run-to-failure tests of bearings are inherently time-consuming and costly. To address these constraints, the experiment was conduct with two bearings in order to have identical operating parameters. This approach minimizes the influence of external variables and enhances data comparability. Nevertheless, additional test and replicates are required to ensure statistical significance. As the assessment of service life involves millions of operating cycles, comprehensive data management presents a significant challenge. Consequently, data collection must be carried out strategically. This dataset contains a limited number of experiments conducted under heterogeneous operating conditions. Data were recorded at regular intervals, therefore changes that may have occurred within a 15-minute interval were not captured. Due to the long duration of the testing, some tests were interrupted to inspect the sensors. The degradation tests were performed at a higher frequency than that experienced by the corresponding components in actual aerospace applications, thereby reducing test duration.

A further limitation lies in defining the point at which a bearing is deemed to be damaged. In this dataset, the precise time of failure was not specified. The measurement records were therefore deliberately continued even after the damage had occurred, in order to record the behaviour during the damage process.

## Ethics Statement

This work does not involve human subjects, animal experiments, or any data collected from social media platforms.

## Credit Author Statement

**Timur Seferli:** Data curation, Writing – original draft preparation; **Jens Windelberg:** Reviewing & editing; **Andreas Bierig:** Reviewing.
